# Bronchiectasis: a bacteriological profile

**DOI:** 10.11604/pamj.2015.22.378.7775

**Published:** 2015-12-17

**Authors:** Régis Gothard Bopaka, Wiam El Khattabi, Hind Janah, Hasna Jabri, Hicham Afif

**Affiliations:** 1 Department of Respiratory Diseases, Hospital on August 20, UHC Ibn Rochd, Casablanca, Morocco

**Keywords:** Bronchiectasis, bacteriological, Streptococcus pneumoniae, pseudomonas aeruginosa, Casablanca

## Abstract

The occurrence of bronchiectasis can involve a combination of many environmental factors, including infection. The aim of our work is to determine the bacteriological profile of bronchiectasis. This is a retrospective study of 100 patients hospitalized in between January 2010 and July 2013. The average age was 48 years with a 58% female predominance. Symptomatology was by a bronchial syndrome in 90% of cases. Bacteriological examination was able to isolate the microbe in 35% of cases. In our study it was through the examination of sputum cytology in 27% of cases, through the examination of liquid bronchial aspiration in 5% of cases, and through direct examination of sputum in search of Mycobacterium tuberculosis in 3% of cases. Microbes isolated were: Streptococcus pneumonia in 11 cases; Pseudomonas aeruginosa in 10 cases, Klebsiella pneumonia and Mycobacterium tuberculosis in 3 cases each; Moraxella catarrhalis, Haemophilus influenzae, Escherichia coli, Citrobacter spp, Serratia marcescens, Mycoplasma pneumoniae, Acinetobacter baumannii and Staphylococcus aureus in one case each. Through this work, the authors highlight that Streptococcus pneumoniae and Pseudomonas aeruginosa are the most commonly- identified microbes in their patients. It is necessary to have a full bacterial examination and to repeat it regularly over the course of the bronchiectasis.

## Introduction

Bronchiectasisis a common illness that is often underestimated. Its treatment is complicated by the fact that it presents clinical and bacteriological heterogeneity. It is a permanent and irreversible increase in the diameter of bronchial tubes from the third to the eight degree. It can be the consequence of many different factors including infection [1]. Bronchiectasis can develop complications which fall into four categories: chronic respiratory failure, hemoptysis, infection and amyloidosis. Infectious complications are the most prevalent, and can come with various bacteriological profiles. Secretions accumulate in the bronchi and cause infections and secondary infections. The goal of this study is to determine the bacteriological profile of this illness as identified in bronchiectasis patients.

## Methods

This study retrospective was conducted between January 2010 and July 2013 with a selection of 100 patients hospitalized with bronchiectasis (patients have a pre-existing diagnosis before hospital admission and during hospitalization). Data was collected according to a premade that gathered personal data, medical history, and symptoms, as well as the results of a chest examination, X-ray, Computerized Tomography scan, and bacteriological examinations. The Computerized Tomography scan diagnosis criteria was a bronchus diameter greater than the associated artery diameter, bronchial tubes visualized at the level of external third of the lung parenchyma and the absence of progressive reduction of bronchial tubes, as they gradually than take away of the hilum [2]. The criteria qualities of the sputum sample were neutrophil higher than 25 elements, and lower than 10 epithelial cells/field, bacteria count upper than 107/ml. The criteria quality of the bronchoaspiration liquid is positive bacteriology (by germs count upper than 10^5^/ml). Data concerning treatment and the evolution of the illness were gathered as well. All data was analyzed using Epi Info Version 6.04. The difference is significant when p <0.05.

## Results

### Patient backgrounds

All patients were of Moroccan origin. 58% of cases were female patients, and 42% were male patients. The average age was 48 years with extremes of 15 and 80 years.

### Relevant medical history

Repeated respiratory infection in childhood was reported in 58% of cases. 35% of patients reported a history of tuberculosis. 2% of patients had Kartagener syndrome.

### Symptoms

Bronchorrhea and purulent sputum was the most prevalent symptom, present in 90% of cases. Dyspnea was present in 76% of cases, and hemoptysis was present in 45% of cases.

### Examination of the chest

Pleuropulmonary auscultation was normal in 25% of cases. Crackles were identified in 60% of cases and wheezing in the remaining 15% of cases.

**Figure 1:**
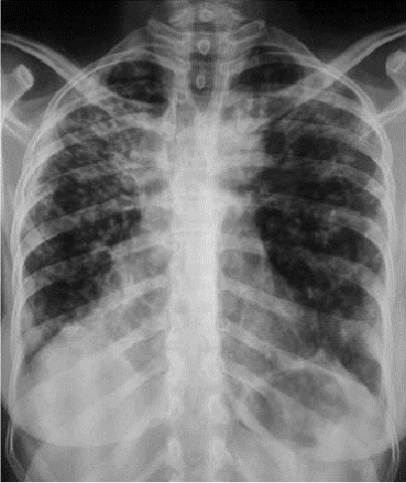
Chest radiograph of a patient with bronchiectasis diffus

**Figure 2:**
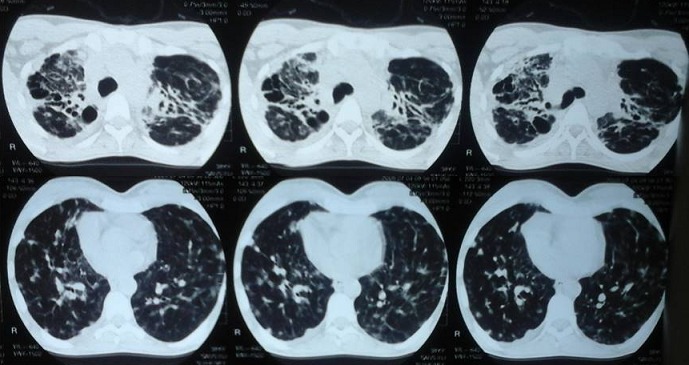
Computerized Tomography scan of patient with bronchiectasis diffus

**Chest radiograph and computerized tomography scan**: (Figure 1 and Figure 2) All cases of Bronchiectasis were confirmed by Computerized tomography scan. The scans identified diffuse lesions in 66% cases and localized lesions in 34% of cases.

### Bacteriological examination

The sampling was performed before antibiotic therapy in 40% of cases and 60% of cases after having received antibiotic therapy before coming to the hospital. The bacteriological examination in all cases was able to isolate the infecting bacteria in 35% of cases (Table 1) and unable to isolate in 65% of cases. The method of successful isolation was examination of sputum cytology in 27 cases, examination of liquid bronchial aspiration in 5 cases, and targeted test for Mycobacterium tuberculosis in sputum in 3 cases.

**Table 1:** Bacteriological profil

Bacteriological	Patients	Percentage
Streptococcus pneumoniae	11	11%
Pseudomonas aeruginosa	10	10%
Klebsiella pneumonia	3	3%
Moraxella catarrhalis	1	1%
Staphylococcus aureus	1	1%
Haemophilus influenzae	1	1%
Escherichia coli	1	1%
Citrobacter spp	1	1%
Acinetobacter baumannii	1	1%
Mycoplasma pneumoniae	1	1%
Serratia marcescens	1	1%
Mycobacterium tuberculosis	3	3%

### Pulmonary function test

Obstructive lung disease was found in 37% of cases. Restrictive lung disease was found in 55% of cases. Pulmonary function test results were normal in the remaining 8% of cases.

### Treatment

In 15% of cases, patients were started on empiric antibiotic therapy which was adapted according to the results of the susceptibility test and the patient's progression (Table 2). Tuberculosis treatment was administered in 3% of cases. Surgery (lobectomy) was performed in 16% of located and complicated bronchiectasis cases. All patients operated had a simple evolution. Bronchial drainage was employed in all cases.

**Table 2:** Bacterial infections and/or bacterial super infections

Bacteriological	Number of cases	Resistance		Sensibility
		Number	Drug	
Streptococcus pneumoniae	11	6	Amoxicillin,Penicillin G	Azitromycin, Claritromycin
Pseudomonas aeruginosa	10	7	Amoxicillin -clavulanic acid,Cefotaxime	Gentamicin,Imipenem
Klebsiella pneumonia	3	2	Amoxicillin -clavulanic acid	Tazobactam,Imipenem
Moraxella catarrhalis	1	1	Amoxicillin, Penicillin G	Azitromycin, Claritromycin
Staphylococcus aureus	1	0	-	-
Haemophilus influenzae	1	1	Amoxicillin -clavulanic acid	Cyprofloxacine
Escherichia coli	1	0	-	-
Citrobacter spp	1	1	Cyprofloxacine	Gentamicin,Imipenem,Sulfamethoxazole-trimethoprim
Acinetobacter baumannii	1	1	Amoxicillin -clavulanic acid	Imipenem
Mycoplasma pneumoniae	1	1	Amoxicillin -clavulanic acid	Erythromycin,Doxycyclin
Serratia marcescens	1	0	-	-
Mycobacterium tuberculosis	3	0	-	-

### Evolution

Chronic respiratory failure developed in 15% of cases. Infection presented in 13% of cases. Only 5% of patients experienced more than 2 flare-ups within the year following the treatment. Favourable evolution was noted in 67% of cases.

## Discussion

The bacteriological profile of diffuse bronchiectasis according to geographical location, the stage of the disease, and the status of the patient immune system. Infections are responsible for oedema of the bronchial mucosa and are the principle elements in Cole's “vicious cycle” [1, 3]. This illness is more likely to affect females, as noted by multiple authors [4, 5]. In our study females represented a higher percentage than males, although the difference was not significant. The average age at which the infection showed in our patients was 48 years, which was lower than that of other studies. Martinez-Garcia MA et al found an average age 69.9 [5], and Darrien found that the infection presented in patients older than 50 years in 75% of cases [6]. The cause of bronchiectasis is difficult to determine, even after an exhaustive examination. The cause of bronchiectasis stays unknown in 50% cases [6]. In our study, the microbe was isolated in only 35% of the cases. The rate of positive bacteriology is relatively low for the hospitalized bronchiectasis cohort. This observation is probably linked to the selection bias inherent in the sampling for 60% of patients received antibiotic therapy before coming to the hospital. The occurrence of bronchiectasis often implicates the conjunction of multiple environmental factors, especially factors that are bacterial in nature. Previous respiratory infections were noted in more than half of patients in the existing literature, but this decreased with the arrival of antibiotics and vaccinations [2, 7]. We found previous childhood respiratory infections in less than 50% of our cases. Tuberculosis can also be responsible for bronchiectasis, as was found in certain cases of our study. Symptomology is not specific for bronchiectasis, as bronchorrhea or dyspnea can be seen in the course of chronic bronchitis [6]. Likewise hemoptysis is not specific to bronchiectasis. An inflammation or infection in the lungs causes this type of hemoptysis [6], however it is one of the greatest causes of bronchiectasis [8]. Normal pleuropulmonary auscultation does not exclude a diagnosis according to this study. The presence of crackles is often evidence of parenchymatous infection and secondary infection. Thecomputed tomography (CT) scan of the chest sets the diagnosis of bronchiectasis. At the same time it allows the doctor to assess extent of the lesions and can indicate an etiological diagnosis. The scan has transformed the diagnostic approach to bronchiectasis particularly as it can detect smaller lesions that pass unseen on a chest x-ray [9–11]. A bacteriological examination must be systematically given to any patient presenting bronchiectasis. This test sometimes makes it possible to isolate the infecting microbe, as 35% of the cases in our study. Some authors have found similar numbers [12], while others have found lower or higher percentages [12, 13] (Table 3). Cytobacteriogical testing of sputum has an important place in this process. In our study this test allowed us to isolate the bacterium in 27% of cases. Examination of liquid bronchial aspiration can also accurately isolate bacterium [14], as was achieved in 5% of cases in our study. Among the bacteria identified, Streptococcus pneumoniae and Pseudomonas aeruginosa were the first and second-most frequently found in our study, respectively 11% and 10% of cases. However, the literature generally presents the most frequently found bacteria as Haemophilus influenzae, Pseudomonas aeruginosa, Staphylococcus aureus, and Streptococcus pneumoniae, in order of decreasing frequency [2]. This difference, especially with regards to Haemophilus influenza, could be due to the relatively younger age of our patients, as Haemophilus influenza is most often found in children and the elderly. Bacteria such as Pseudomonas aeruginosa can grow into a colony within their hosts. The presence of a Pseudomonas aeruginosa colony is correlated with decreased quality of life [15, 16]. It is an independent factor associated with an accelerated decline in respiratory function [5]. It presents in later stages of bronchiectasis and marks a downward turning point in the progression of the disease [5, 16–18]. The testing of sputum for Koch's bacillus must be performed. Mycobacterium tuberculosis was found in 3 patients (3%) in our study, while other authors found as many as 9 cases in a study of 91 patients with bronchiectasis [19]. The progression and prognosis for bronchiectasis are a function of how widespread the lesions are and the medical history of the patient [6]. Chronic respiratory failure is the most reliable evidence of bronchiectasis that has been developing over the course of several years.

**Table 3:** Comparing data between the study publications and our study

Bacteriological	Our study	Wilson CB et al [12]	Hare KM et al [13]
Streptococcus pneumoniae	11%	5,7%	-
Pseudomonas aeruginosa	10%	13,7%	-
Klebsiella pneumonia	3%	-	-
Moraxella catarrhalis	1%	3,4%	-
Staphylococcus aureus	1%	4,5%	-
Haemophilus influenzae	1%	19,5%	39%
Escherichia coli	1%	-	-
Citrobacter spp	1%	-	-
Acinetobacter baumannii	1%	-	-
Mycoplasma pneumoniae	1%	-	-
Serratia marcescens	1%	-	-
Mycobacterium tuberculosis	3%	-	-

## Conclusion

Through this work, the authors emphasize that the bacteriological profile of bronchiectasis is variable. Streptococcus pneumoniae and Pseudomonas aeruginosa were the most commonly isolated bacteria in their patients. The presence of Pseudomonas aeruginosa has a poor prognosis. Regular and repeated bacterial checkups are necessary over the course of bronchiectasis.
